# Interacting with the biomolecular solvent accessible surface via a haptic feedback device

**DOI:** 10.1186/1472-6807-9-69

**Published:** 2009-10-27

**Authors:** Matthew B Stocks, Steven Hayward, Stephen D Laycock

**Affiliations:** 1 School of Computing Sciences, University of East Anglia, Norwich, UK, NR4 7TJ; 2 School of Biological Sciences, University of East Anglia, Norwich, UK, NR4 7TJ

## Abstract

**Background:**

From the 1950s computer based renderings of molecules have been produced to aid researchers in their understanding of biomolecular structure and function. A major consideration for any molecular graphics software is the ability to visualise the three dimensional structure of the molecule. Traditionally, this was accomplished via stereoscopic pairs of images and later realised with three dimensional display technologies. Using a haptic feedback device in combination with molecular graphics has the potential to enhance three dimensional visualisation. Although haptic feedback devices have been used to feel the interaction forces during molecular docking they have not been used explicitly as an aid to visualisation.

**Results:**

A haptic rendering application for biomolecular visualisation has been developed that allows the user to gain three-dimensional awareness of the shape of a biomolecule. By using a water molecule as the probe, modelled as an oxygen atom having hard-sphere interactions with the biomolecule, the process of exploration has the further benefit of being able to determine regions on the molecular surface that are accessible to the solvent. This gives insight into how awkward it is for a water molecule to gain access to or escape from channels and cavities, indicating possible entropic bottlenecks. In the case of liver alcohol dehydrogenase bound to the inhibitor SAD, it was found that there is a channel just wide enough for a single water molecule to pass through. Placing the probe coincident with crystallographic water molecules suggests that they are sometimes located within small pockets that provide a sterically stable environment irrespective of hydrogen bonding considerations.

**Conclusion:**

By using the software, named HaptiMol ISAS (available from ), one can explore the accessible surface of biomolecules using a three-dimensional input device to gain insights into the shape and water accessibility of the biomolecular surface that cannot be so easily attained using conventional molecular graphics software.

## Background

The sense of touch can be used to augment our visual sense to gain a deeper insight into the three dimensional shapes of complex objects. Biomolecules are examples of highly complex three dimensional objects which are often visualised using molecular graphics. Many software programs exist which attempt to convey the three dimensional form of structures utilising stereoscopic viewing methods and depth cues. However, the augmentation of our sense of sight with touch would be a useful aid in understanding the overall three dimensional shape of a biomolecule and in particular the fine surface details that cannot easily be seen whilst visualising the molecule as a whole. With what should one "touch" a biomolecule?

Intuitively a sphere seems to be an obvious choice. Fortuitously, hard-sphere interactions between the biomolecule and a sphere of radius equal to an oxygen atom provides a reasonable model of solvent-solute interaction. Thus touching the biomolecular surface with a sphere the size of a water molecule one could also determine solvent-accessible regions of the biomolecule [1,2]. Modelling touch requires a force-feedback or haptic device capable of exerting forces on the user. The process of determining the forces transmitted to the haptic device is known as haptic rendering and in this case works by computing forces of interaction between the probe, and the simulated biomolecule. This allows the user to feel the combined force acting on the probe.

In the area of biomolecular research the probe is usually a small molecule, known to interact with the biomolecule, where the forces are due to electrostatic and van-der-Waals interactions. The application of haptics to "molecular docking" has quite a long history with the first such project, GROPE1, starting in 1967 at the University of Carolina [3]. Similar but more recent applications using personal computers allow the user to feel electrostatic forces between the probe molecule and the biomolecule [4,5]. Up until recently these applications always assumed that both the protein and the ligand are completely rigid. However, "Interactive Molecular Dynamics" (IMD), [6] includes molecular flexibility by allowing the user to apply forces through a haptic device during a Molecular Dynamics simulation.

Our approach is quite different to previous applications of haptic rendering in the area of biomolecular simulation in that we aim to provide the user with a deeper appreciation of the complex three-dimensional shape of the molecule by combining a variety of graphical rendering techniques with haptic interactions. In the software a sphere, with a user-specified radius, is manipulated to interact with the chosen biomolecule. By using a probe sphere that is the same size as a water molecule, hard-sphere interactions with the biomolecule can be calculated to determine regions on the molecular surface that are accessible to water. In that sense our application could be called "Interactive Solvent-Accessible Surface" (ISAS).

## Implementation

**Figure 1:**
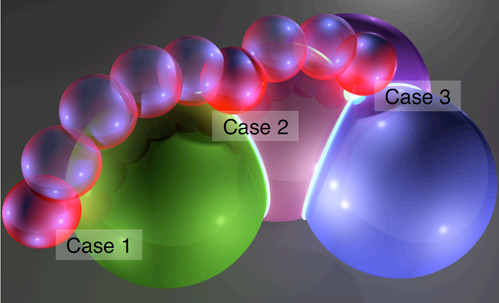
**The main features used in the surface tracking algorithm**. An illustration of how the algorithm allows for proxy based haptic rendering of a molecular surface represented in space filling form. The three main features of a space filling molecule are marked as follows: Case 1: The HIP has penetrated a single sphere so the SCP can be calculated as the closest point on the surface, Case 2: The HIP is located between two intersecting atoms therefore the SCP must now be calculated as the closest point on the circle of intersection, Case 3: The HIP is situated beneath the intersection of several atoms. The intersection forms single points which can be calculated and used as the current position of the SCP, when the HIP moves out of this area the algorithm will then choose the next feature to calculate the new position of the SCP.

In this work molecules are represented as a space filling CPK model, with each atom defined as a separate sphere. In order to touch the biomolecular structure a haptic rendering algorithm must be created to compute forces as the user manipulates the Haptic Interface Point, HIP. The HIP is a single three dimensional coordinate defining the location of the end point of the haptic stylus within the virtual environment. A typical constraint-based single point haptic rendering algorithm involves approximating the surfaces of the objects in the virtual environment by polygonal meshes [7,8]. For the haptic rendering of molecules, approximating each atom by a polygonal surface is inefficient and leads to undesirable discontinuities in the force feedback. The algorithm used here [9], alleviates the need for this approximation by exploiting the spherical shapes and overlapping configurations that naturally occur in the space filling representation. Once the HIP has penetrated an atom, a surface contact point, SCP, is set to the closest point on the surface of the sphere representing the atom. The basic approach is then to track the SCP over the features of the molecule as the user manipulates the HIP. At any given point the SCP is in contact with one of the following molecule features: a single sphere (Case 1), an intersection circle formed when two spheres intersect (Case 2), the intersection point between multiple spheres (Case 3). The force returned is based on the spring between the HIP and the SCP. Figure 1 illustrates the three stages of the algorithm employed, further details can be obtained in the paper by Stocks and Laycock [9].

To allow a probe sphere to be used with a user-specified radius the above single point algorithm need not be modified. Instead, the van der Waals radius of every atom is enlarged by the radius of the probe sphere. The single point haptic rendering algorithm then utilises the enlarged representation whilst the spherical probe and original molecule are displayed to the user. To achieve the interactive accessible surface simulation a probe sphere with a radius equal to the van der Waals radius of an oxygen atom can be created and centered at the location of the HIP. The force returned through the haptic device is designed to mimic the resulting hard sphere interactions between the probe and the contacting atoms on the biomolecule. The effect is to see and feel the water probe roll around over the hard surface of the biomolecule. In order to visually guide the user to regions on the biomolecular surface known to bind water molecules, crystallographic water molecules are rendered graphically but not haptically meaning they can be seen but not felt. Crystallographic water molecules are visualized as semi-opaque spheres through the use of alpha blending. These are referred to as "ghost water". To guide the user to specific residues or ligands the user can select from a variety of colours not contained within the CPK colour system and assign these to residues selected by residue number and chain identifier.

**Figure 2:**
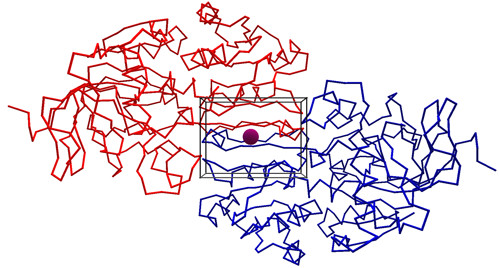
**A backbone trace rendering of liver alcohol dehydrogenase**. The backbone trace can be visualised to enable the user to orientate the molecule as desired. The navigation cube is still visible because the haptic device can be used exactly as if the space filling representation were shown. However, the user will feel no force feedback until returning to a space filling representation. Here the individual subunits are coloured red and blue.

**Figure 3:**
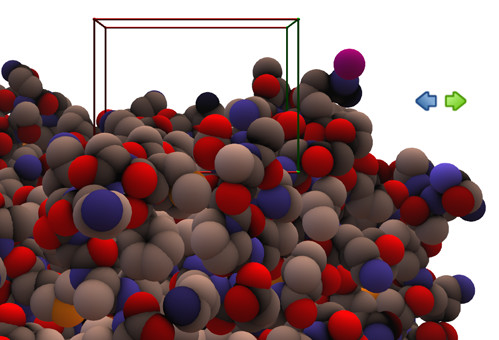
**Translating the protein using the haptic device**. The user is presented with a navigation cube which visualises the boundaries of the safe working area of the haptic device being used. Moving the device out from the edges of the cube will translate the molecule so that the desired area of the protein can be safely studied. As the user moves the device out from the green edge of the cube in the direction of the green arrow the protein will start to translate in the direction of the blue arrow on the image.

**Figure 4:**
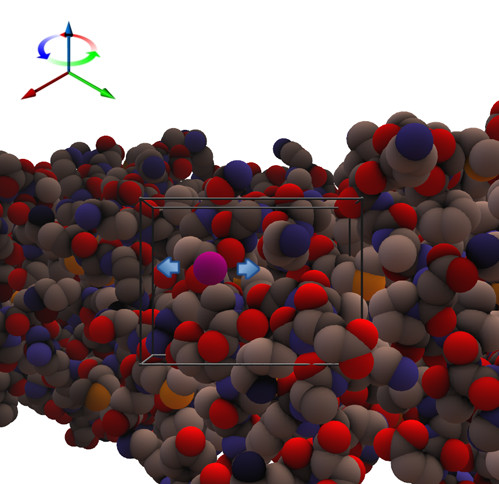
**Rotating the protein using the haptic device**. While inside the navigation cube, the user can hold the front button on the haptic stylus to allow rotation of the protein. As the user holds the button on the stylus and moves the device in the direction of either blue arrow shown in the image, a rotation around the blue axis, as shown in the top right of the image, will occur.

**Figure 5:**
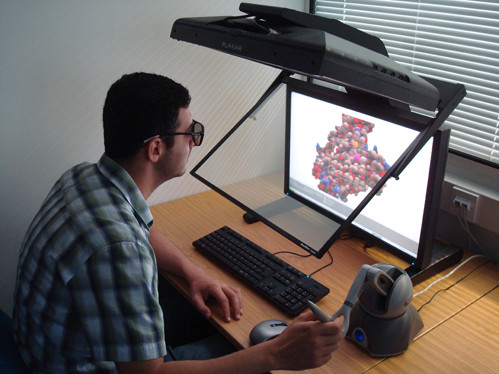
**Viewing 3D stereoscopic molecules in conjunction with haptics for exploration**. To enhance the three dimensional view of the molecule typical strategies such as shadows, depth cueing and stereoscopic views have been incorporated. The optional stereoscopic view is based on a quad buffer technique and therefore requires a compatible graphics card, such as an NVidia Quadro, and appropriate 3D display technology, such as the PLANAR's widescreen 26" Stereo monitor.

To help the user orientate the molecule the backbone trace can be displayed (it is not haptically rendered), as depicted in Figure 2. A unique feature of the software is in the way the haptic device is used not only to touch the molecule but also to rotate and translate it. As the user explores a large structure, the software translates the molecule automatically to centre the view to the probe location. This removes the difficulty of using a mouse for navigation in combination with the haptic device. A video demonstrating this can be found at the following location: . This approach works by visualising the region of the molecule which may be explored haptically as a cube, termed the "navigation cube". To explore areas of the molecule outside this cube, the user simply moves the probe sphere in the direction of a new location, as depicted in Figure 3. At this point the molecule will automatically translate to enable the new location to be reached. To rotate the molecule the user can hold down the button on the haptic stylus and move it in the direction of the desired rotation, as shown in Figure 4. The navigation cube is similar to the bubble technique [10], however, the spherical navigation volume in that approach does not correspond well to the workspace of the haptic device, whereas here the navigation cube is automatically scaled to fit the workspace. Furthermore, unlike the bubble technique no forces of interaction between the navigation cube and the probe are included to avoid confusion with forces arising due to interaction with the biomolecule. To indicate the location of the probe when it is occluded, a "2D cursor" can be used to indicate the location of the probe in the x-y plane. To enhance the three dimensional view of the molecule typical strategies such as shadows, depth cueing and stereoscopic views have been incorporated. The optional stereoscopic view is based on a quad buffer technique and therefore requires a compatible graphics card, such as an NVidia Quadro, and an appropriate 3D display technology. A typical 3D display being utilised is depicted in Figure 5.

## Results

### Liver Alcohol Dehydrogenase

**Figure 6:**
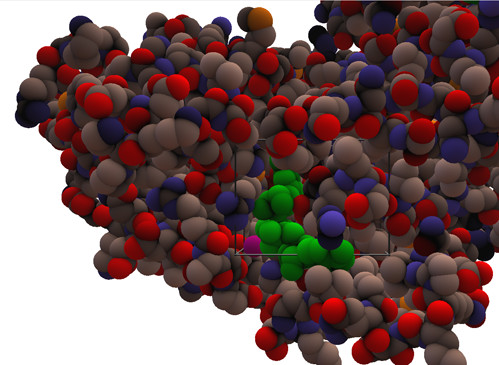
**Liver alcohol dehydrogenase with the inhibitor SAD**. The selection tool that can be used to highlight regions of interest in the protein is illustrated. In this case the ligand, SAD, is coloured green. The probe, in magenta, is seen emerging from the interdomain crevice next to SAD and Arg47.

LADH provides an interesting test case as it has an interdomain crevice. The binding of the coenzyme NAD to the coenzyme-binding domain causes the domains to close at which point water must move out of the interdomain crevice. An open form of LADH (PDB: 1ADG) which is bound to SAD was used [11]. SAD is a compound that is unable to induce closure as it lacks the nicotinamide group of NAD [12]. The other structure analysed was a closed form of LADH bound to NAD (PDB: 2OHX) [13]. In the open structure the water probe was moved into the interdomain crevice at Pro296 and was able to move through a narrow channel past SAD to emerge at Arg47 (see Figure 6). In places guidance of the probe is helped by the presence of the ghost water. At its narrowest, the channel is clearly only wide enough for one water molecule. The path from one side to the other is not direct, and occasionally when trying to manoeuvre the water molecule it becomes caught in an interior pocket formed by residues 48,67,93,116,141 and 376. In the closed structure this passage is closed due to the presence of NAD's nicotinamide group and the further narrowing of the passage due to domain closure. These details would be difficult to determine from existing software which only offer a visual representation of the accessible surface. A video of this process can be seen at the following location: .

### Acetylcholinesterase

**Figure 7:**
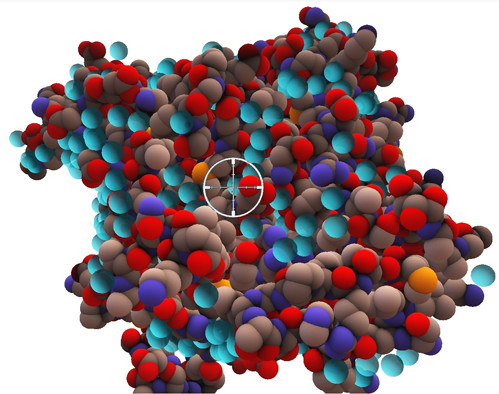
**The active site cavity in acetylcholinesterase**. The use of the haptic device to study the deep active site cavity in acetylcholinesterase is illustrated. The probe is in the cavity as indicated by the 2D cursor (depicted as a circle with cross-wires) that gives the probe's x-y position when it is occluded. This allows the user to view the whole molecule whilst gaining an appreciation of the shape and extent of the cavity through their sense of touch by moving the probe around. Cystallographic water molecules are depicted as semi-opaque spheres in cyan. These are seen but not felt, thus the term "ghost water".

Acetylcholinesterase is found in nerve synapses and breaks down acetylcholine into its two component parts, acetic acid and choline. It was selected as its active site is buried deep in a cavity that is accessible to water. The high-resolution structure bound to the nerve-agent O-ethyl-S- [2-[bis(1-methylethyl)amino]ethyl] methylphosphonothioate (VX) with PDB accession code 1VXR was selected [14]. Figure 7 shows the structure surrounded by crystallographic water molecules where the probe is deep inside the active site cavity. To indicate the location of the probe in this case the 2D Cursor was used, as shown in the figure. During this interaction an impression of the molecule that is not possible through the use of conventional molecular graphics is obtained, as at the same time as gaining an overall view of the protein, through one's sense of touch one can simultaneously get an impression of the size and shape of the cavity by moving the probe around within it.

**Figure 8:**
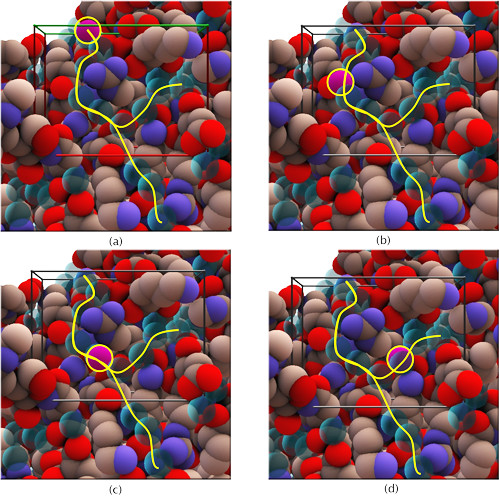
**Crystallographic water molecules on the protein surface, positions (a-d)**. A network of crystallographic water molecules in acetylcholinesterase is indicated by the "Y" shaped curve in yellow. These water molecules are centred on Arg220 which can be seen close to the "valley" of the "Y". The probe was moved over the surface placing it at the crystallographic water locations (four of which are indicated in the figure by a yellow circle), each of which gave the impression through the haptic device of being locally stable.

Figure 8 shows a close up on the surface of the acetylcholinesterase molecule centred on Arg220 showing a "Y" shaped network of closely neighbouring crystallographic water molecules. The probe can be positioned at each water molecule location as they are not rendered haptically (ghost water). By placing the probe coincident with crystallographic water molecules one feels a hindrance to the probe's movement over the surface of the protein as if the probe is partially trapped. This seems to suggest that these water molecules are situated at sites that provide a stable steric environment, irrespective of any hydrogen bonding considerations.

## Discussion

A haptic rendering application for biomolecular visualisation has been developed that allows one to gain three-dimensional awareness of the shape of a biomolecule. By using a water molecule as the probe, the process of exploration has the further benefit of being able to determine regions on the molecular surface that are accessible to the solvent. Aside from the simultaneous three-dimensional insight into the shape of the molecule, what other advantage would this method have over standard solvent-accessible surface area calculations? One obvious advantage is that one can easily appreciate the dimensions of a channel and the number of water molecules that would fit through it. Another advantage is that the accessibility of a channel or cavity can be appreciated. A cavity with an opening where the user has difficulty in maneuvering a water molecule could indicate an entropic bottleneck. In addition, placing the probe coincident with crystallographic water molecules gave the distinct impression that these are located within small pockets that provide a sterically stable environment for water molecules. None of this information would be directly attainable from standard solvent-accessible surface area calculations. It is clear that our approach has the limitation of not accurately modelling interactions and response due to flexibility that occurs when a water molecule approaches a biomolecule. Reduction of the probe radius could be used to model the effects of flexibility in a simple way. A better alternative would be to use the tool to compare accessilibility to channels and cavities in conformations generated from Molecular Dynamics simulations.

## Conclusion

Haptic rendering combined with molecular graphics allows the user to feel as well as see a complex three-dimensional object. In its application to biomolecular modelling it allows one to not only gain insight into the shape of a biomolecule, but by using a spherical probe equivalent in size to a water molecule, it also allows one to explore the solvent accessible surface interactively by rolling the probe over the molecule. Although many of the insights into cavity shape may be gained by purely graphical techniques, usage of the system has shown that it allows the user to assess the difficulty water molecules may have in accessing or escaping a cavity through the difficulty the user has in manoeuvring the probe through a constriction.

This would not be easily appreciated through purely graphical techniques.

## Availability and Requirements

• **Project Name**: HaptiMol ISAS

• **Project Home Page**: 

• **Operating System(s)**: Windows 2000, XP, Vista 32bit, Vista 64bit

• **Programming Language**: C++

• **Other Requirements**: OpenGL version 2.0 or later is required for high quality rendering. However, if a lower version is detected the program will adjust the rendering algorithm accordingly. The software supports all Phantom Haptic Feedback Devices (Phantom Omni, Phantom Premium, Phantom Premium 6DOF and Phantom Desktop). OpenHaptics Software (Academic Edition) is available for free download.

• **License**: Free

• **Any restrictions to use by non-academics**: The current release is for non-commercial use only.

## List of Abbreviations Used

HIP: Haptic Interface Point; The virtual end point of the haptic device, this point is not visible to the user. SCP: Surface Contact Point; The point calculated in the surface tracking algorithm, this point is visible to the user. LADH: Horse Liver Alcohol Dehydrogenase; SAD: *β*-methylene-selenazole-4-2 carboxyamide-adenine dinucleotide; NAD: nicotinamide adenine dinucleotide.

## Authors' contributions

MBS and SDL designed and implemented the haptic rendering algorithms and software detailed in this paper. SH designed the requirements for the software taking into account the current limitations of software already available. SH selected the test cases, demonstrating the utility of the software, and coordinated user testing with other structural biologists. Each author drafted the part of the manuscript most directly related to them. All authors read and approved the final manuscript.
